# Haspin balances the ratio of asymmetric cell division through Wnt5a and regulates cell fate decisions in mouse embryonic stem cells

**DOI:** 10.1038/s41420-023-01604-w

**Published:** 2023-08-23

**Authors:** Yingying Gao, Bin Ma, Yifan Li, Xiangyu Wu, Shifeng Zhao, Huiping Guo, Yiwei Wang, Lihua Sun, Jing Xie

**Affiliations:** 1 Fundamental Research Center, Shanghai Yangzhi Rehabilitation Hospital (Shanghai Sunshine Rehabilitation Center), Frontier Science Center for Stem Cell Research, School of Life Sciences and Technology, Tongji University, Shanghai, 200092 China; 2 Reproductive Medicine Center, Shanghai East Hospital, School of Medicine, Tongji University, Shanghai, 200092 China; 3 Shanghai Key Laboratory of Maternal Fetal Medicine, Shanghai Institute of Maternal-Fetal Medicine and Gynecologic Oncology, Shanghai First Maternity and Infant Hospital, School of Medicine, Tongji University, Shanghai, 200092 China; 4 Present Address: Department of Biology, Johns Hopkins University, Baltimore, MD 21218 USA

**Keywords:** Embryonic germ cells, Stem-cell differentiation

## Abstract

Many different types of stem cells utilize asymmetric cell division (ACD) to produce two daughter cells with distinct fates. Haspin-catalyzed phosphorylation of histone H3 at Thr3 (H3T3ph) plays important roles during mitosis, including ACD in stem cells. However, whether and how Haspin functions in ACD regulation remains unclear. Here, we report that Haspin knockout (Haspin-KO) mouse embryonic stem cells (mESCs) had increased ratio of ACD, which cumulatively regulates cell fate decisions. Furthermore, Wnt5a is significantly downregulated due to decreased Pax2 in Haspin-KO mESCs. Wnt5a knockdown mESCs phenocopied Haspin-KO cells while overexpression of Wnt5a in Haspin-KO cells rescued disproportionated ACD. Collectively, Haspin is indispensable for mESCs to maintain a balanced ratio of ACD, which is essential for normal development and homeostasis.

## Introduction

Stem cells have a unique ability to both self-renew and generate daughter cells capable of differentiating. Many different types of stem cells utilize asymmetric cell division (ACD) to produce two daughter cells with distinct fates: one stem cell and one differentiating cell [1–5]. Alternatively, stem cells can also undergo proliferating symmetric cell divisions (SCD) which produce two identical daughters. ACD must be rigorously controlled to prevent excessive proliferation or differentiation which can lead to diseases ranging from cancer to tissue degeneration [4, 6]. Hence, a balance between these two forms of division is essential for normal development and tissue homeostasis.

Stem cells evolved a variety of cellular and epigenetic mechanisms to regulate a proper ratio of ACD, and tiny variations can lead to total disruption of this balance. For instance, in *Drosophila* male germline stem cells (GSCs), transient phosphorylation of Histone H3 at Thr3 (H3T3ph) was found to be a pivotal mark to distinguish old versus new H3-enriched sister chromatids in the prophase of ACD. Notably, overexpression of two H3 mutants, H3T3A or H3T3D, which perturbed H3T3ph in a dominant way, disrupted the ACD ratio among the GSCs. As a consequence, this leads to either loss of germline stem cells or germline tumors [7], indicating that H3T3ph may play an integral role in ACD regulation in stem cells.

Haspin is the only known kinase for H3T3ph [8]. It was initially identified in mouse testicular germ cells and is encoded by germ cell-specific gene 2 (Gsg2) [9, 10]. Haspin has been found to be tightly regulated in a temporal-specific manner as H3T3ph can only be detected during mitosis from the onset of prophase to anaphase across multiple species. Haspin is inactive during interphase and becomes active to catalyze H3T3ph when the cell entering mitosis [11]. Then H3T3ph serves as a chromatin-binding site for chromosomal passenger complex (CPC) to inner centromeres. There, via its subunit Survivin, CPC works to ensure correct chromosome separation during mitosis [12–14]. Haspin RNA interference causes low levels of H3T3ph and misalignment of metaphase chromosomes [8, 15]. Moreover, Haspin depletion leads to activation of the spindle checkpoint, arresting mitosis in a prometaphase-like state [13], while overexpression of Haspin results in a delay prior to metaphase [8, 16].

In addition to be the “writer” of H3T3ph, Haspin has been reported to possess non-canonical functions. For example, it could help proper sister-chromatid cohesion by binding to Pds5B [17], and antagonize Wapl-mediated cohesion release from mitotic centromeres [18]. Recently, Soupsana and colleagues found that Haspin is not essential for both pluripotency and differentiation of ESCs [19, 20]. However, it remains unclear whether (and how) Haspin is directly involved in the regulation of ACD, and consequentially, influences the cell fate decisions during the lineage developments of stem cells.

In this study, aiming to understand Haspin’s role in the ACD of stem cells and consequential impacts on cell fate decisions, we generated Haspin functional knockout (Haspin-KO) mouse embryonic stem cells (mESCs) with CRISPR/Cas9 and quantified the ratio of ACD in these cells. Moreover, we investigated its cumulative effects on lineage development after differentiation induction. We also performed RNA-seq to look for downstream factors, trying to reveal the mechanisms underlying how Haspin maintains a balanced ratio of ACD in stem cells, which is essential for normal development and homeostasis.

## Results

### Construction of Haspin functional knockout mouse embryonic stem cell lines

**Fig. 1:**
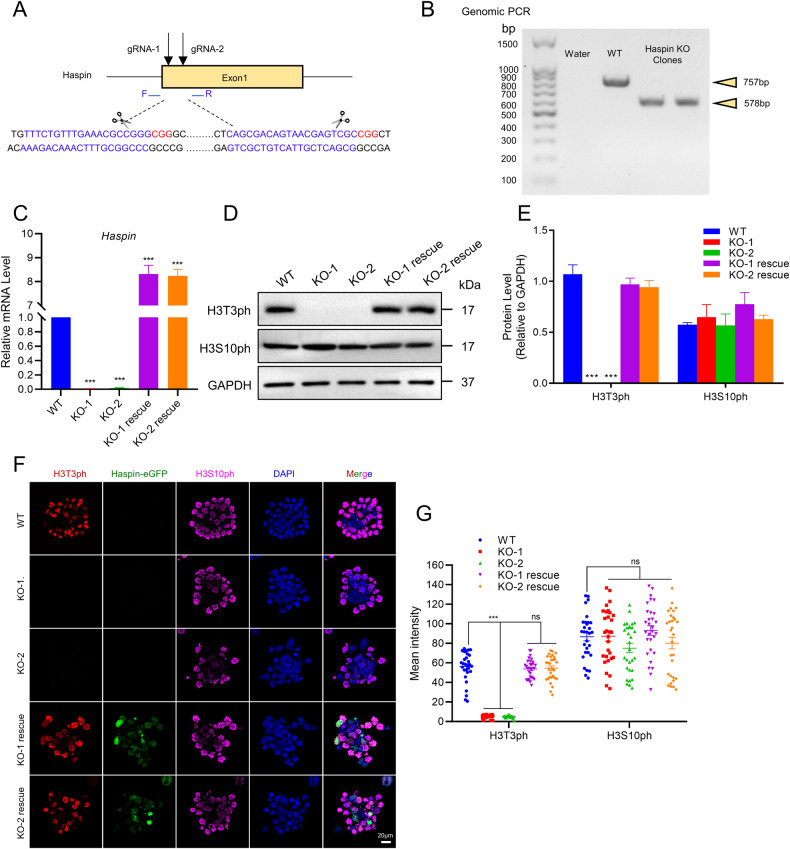
Generation of Haspin-KO cell lines through CRISPR/Cas9. **A** Schematic illustration of the creation of Haspin-KO mESCs through CRISPR/Cas9 technology. The blue sequences are gRNA sequences, and the red sequences are PAM sequences. F and R primers were used to perform genomic PCR. **B** Genomic PCR for identifying Haspin knockout. **C** qRT‒PCR analysis of *Haspin* expression levels in WT, Haspin-KO, and rescued cells. **D** Western blotting analysis of H3T3ph and H3S10ph in WT, Haspin-KO, and rescued cells. **E** The statistical results of protein expression in (**D**) (*n* = 3). **F** Immunofluorescence of H3T3ph and H3S10ph in WT, Haspin-KO, and Haspin-eGFP overexpressing (rescue) cells. Scale bar: 20 μm. **G** The statistical results of the fluorescence intensity of (**F**). *n* = 30 from three independent experiments. The data in (**C**), (**E**), and (**G**) are represented as the mean ± SEM. *p < 0.05, **p < 0.01 and ***p < 0.001.

To uncover the potential role of Haspin in mESCs, we generated Haspin-KO mESC lines using CRISPR/Cas9 technology (Fig. 1A). Genome PCR and quantitative RT‒PCR (qRT‒PCR) were performed to prove the loss of Haspin at both the genomic and mRNA levels in the two Haspin-KO mESC lines (Fig. 1B, C). We also generated cell lines expressing Haspin-eGFP in Haspin-KO mESCs for rescue experiments. As a read out for Haspin kinase activity, H3T3ph level was probed to indicate functional knock out of Haspin [8]. Besides, phosphorylation of Histone H3 at Ser10 (H3S10ph), another transient H3 phosphorylation in mitosis, was examined as a control [21]. Both western blotting and immunofluorescence showed abolishment of H3T3ph in these two independent Haspin-KO mESCs lines, which could be restored by the rescue constructs (Fig. 1D–G).

In considering of Haspin’s important role in mitosis, we examined the effect of Haspin-KO on the progression of cell cycle and proliferation in mESCs. Although Haspin-KO cells grew more slowly than WT cells, the cell cycle was not impaired in Haspin-KO mESCs (Fig. S1A, B). Since cleaved caspase-3 was a marker for cell apoptosis [22], we detected the effect of Haspin-KO on protein levels of cleaved caspase-3 and caspase-3 by western blotting. Notably, Haspin-KO significantly increased the expression of cleaved caspase-3 and induced cell apoptosis (Fig. S1C, D). These data together suggest that lack of Haspin may increase the apoptosis ratio among the cells and the slower growth of Haspin-KO cells we observed is partly because of apoptosis. In sum, these results indicate that both Haspin-KO mESC lines and rescued lines are qualified for follow-up experiments.

### Haspin-KO mESCs increase ACD ratio and lead to differentiation defects

**Fig. 2:**
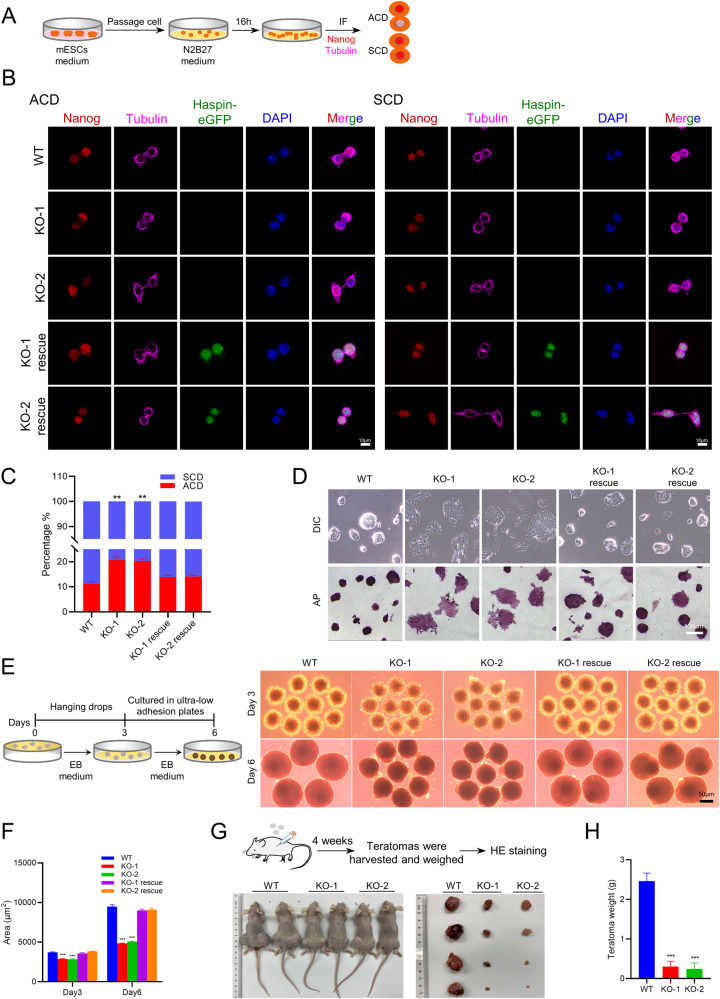
Haspin-KO increases the ratio of ACD and causes defective development capacity. **A** Schematic illustration of the steps for the induction of asymmetric stem cell division (ACD). **B** Representative images of WT, Haspin-KO, and rescued mESCs cultured with N2B27 medium and stained with Nanog and Tubulin antibodies. Scale bar: 10 μm. ACD: asymmetric stem cell division, SCD: symmetric stem cell division. **C** The percentages of ACD and SCD were defined by Nanog fluorescence intensity in WT, Haspin-KO, and rescued mESCs. *n* = 150 from three independent experiments. **D** Colony morphology and AP staining of WT and Haspin-KO and rescued mESCs. Scale bar: 100 μm. **E** Schematic illustration of the steps for embryoid body (EB) formation. Phase-contrast microscopy images of EBs obtained from mESCs on Day 3 and Day 6. Scale bar: 50 μm. **F** The area statistics of EB. *n* = 20 for Day 3 and Day 6. **G** Schematic illustration of the teratoma formation and tissue analysis. Teratomas formed from nude mice subcutaneously injected with WT or Haspin-KO mESCs at 4 weeks. **H** The statistical analysis of teratoma weights (*n* = 4). The data in (**C**), (**F**), and (**H**) are represented as the mean ± SEM. *p < 0.05, **p < 0.01 and ***p < 0.001.

Previous studies found that transient H3T3ph played a vital role in *Drosophila* male GSC for proper ACD. Once phosphorylation at the H3T3 site was perturbed by expression of H3T3A/D, the ACD ratio in GSCs was disturbed [7]. Therefore, we wondered whether Haspin could directly regulate ACD in mESCs. To identify the two daughter cells that came from one mother, we utilized tubulin-labeled intercellular bridges as a mark for the paired cells [23]. To determine the ratios of ACD, we used similar method as described in the previous studies [24–26], indicated by Nanog expression between two daughter cells (Fig. 2A). Consistent with previous report [24], the ACD ratio in WT cells was ~11%. Interestingly, the ACD ratio significantly increased in the Haspin-KO mESCs and rebalanced in the rescue group of Haspin-eGFP expressing cells (Fig. 2B, C), suggesting Haspin influences the ACD ratio directly in mESCs.

Furthermore, in Haspin-KO mESCs, significant morphological changes were observed, including flattened colonies and weaker alkaline phosphatase (AP) staining, which were recovered by the rescue constructs (Fig. 2D). Similar phenotypes were observed in Haspin knockdown (KD) mESCs by siRNA (Fig. S2E). Haspin KD efficiency was verified by qRT‒PCR, western blotting, and immunofluorescence (Fig. S2A–D). Then, we detected the expression of the core pluripotent factors including Nanog, Oct4, Sox2, and Klf4 in Haspin-KO mESCs. We didn’t see altered expression of Nanog, Oct4, and Sox2, though Klf4 was slightly downregulated (Fig. S3A, B). Notably, although Haspin-KO did not affect the expression of key pluripotency markers, it led to disordered expression of differentiation genes (Fig. S2F, G). These results show that Haspin does not play a major role in pluripotency in mESCs.

To further investigate the influences of Haspin-KO in differentiation, we used embryoid body (EB) induction and teratoma formation assays to test the differentiation capability of Haspin-KO mESCs both in vitro (Fig. 2E) and in vivo (Fig. 2G). Haspin-KO mESCs produced EBs of markedly reduced size compared with WT cells did, and such defect was rescued by overexpression of Haspin-eGFP (Fig. 2E, F). In addition, qRT‒PCR analysis showed that the expression levels of three germ layer markers are elevated in Haspin-KO EBs (*Nestin* and *Fgf5* as ectoderm markers, *Brachyury* and *T* as mesoderm markers, *Gata4* and *Gata6* as endoderm markers) (Fig. S3C). Notably, overexpression of Haspin-eGFP rescued this disorder (Fig. S3C). Similar to the Haspin-KO mESCs results, protein expression of cleaved caspase-3 was also significantly elevated in Haspin-KO cells formed EBs on Day 6 (Fig. S3D, E). Consistently, teratomas derived from Haspin-KO mESCs in vivo exhibited significantly reduced weight and size compared with the WT control (Fig. 2G, H). As shown by H&E histological analysis, WT cell-derived teratomas comprised three germ layers with normal morphology, including neural rosettes and epithelium (ectoderm), muscle and cartilage (mesoderm), and intestinal epithelium (endoderm). However, despite that all three germ layers could be observed in Haspin-KO mESC-induced teratomas, their tissue types were reduced, and the degree of differentiation was decreased (Fig. S3F). What’s more, qRT‒PCR analysis showed mRNA levels of all three embryonic germ layer markers were disordered in Haspin-KO teratomas (Fig. S3G).

Taken together, deficiency of Haspin caused an increased ratio of ACD and multiple defects during stem cell lineage differentiations, which all can be rescued by the replenish of Haspin-eGFP. Thus, Haspin is indispensable for mESCs to maintain a balanced ratio of ACD which is essential for normal development and homeostasis.

### Haspin kinase activity is indispensable to regulate the ACD ratio in mESCs

**Fig. 3:**
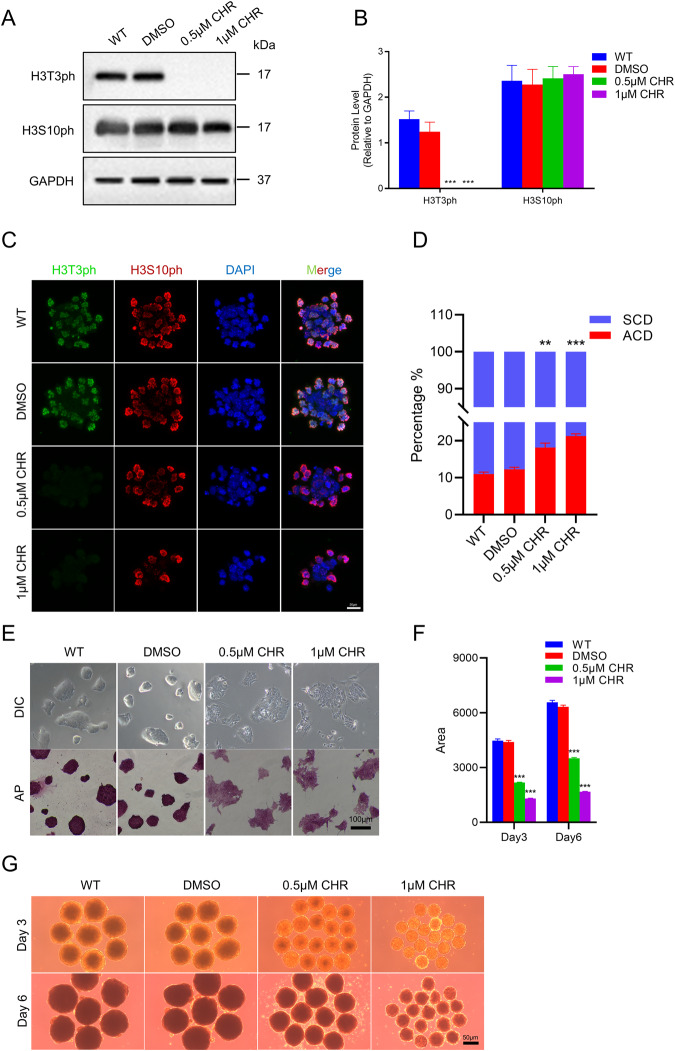
Effect of CHR-6494 on ACD and developmental potencies. **A** Protein levels of H3T3ph and H3S10ph in WT, DMSO, 0.5 μM CHR-6494 and 1 μM CHR-6494 treated mESCs. **B** The statistical results of protein expression in (**A**) (*n* = 3). **C** Immunofluorescence of WT, DMSO, 0.5 μM CHR-6494, and 1 μM CHR-6494 treated cells for H3T3ph and H3S10ph. Scale bar: 20 μm. **D** The statistical analysis of ACD and SCD in WT, DMSO, 0.5 μM CHR-6494 and 1 μM CHR-6494 treated mESCs. *n* ≈ 80 from three independent experiments. **E** Representative images of colony morphology and AP staining in WT, DMSO, and CHR-6494 treated cells. Scale bar: 100 μm. **F** The statistical analysis of the EB area in WT, DMSO, and CHR-6494 treated cells. *n* = 20 for Day 3 and Day 6. **G** Representative images of EB were obtained from WT, DMSO, and CHR-6494 treated mESCs at Day 3 and Day 6. Scale bar: 50 μm. The data in (**B**), (**D**), and (**F**) are represented as the mean ± SEM. *p < 0.05, **p < 0.01 and ***p < 0.001.

To further determine whether the kinase activity of Haspin is essential for ACD regulation, we blocked Haspin kinase with its specific inhibitor, CHR-6494 [27] and quantified the ratio of ACD in WT mESCs. Western blotting and immunofluorescence of H3T3ph were performed to verify the inhibition of Haspin kinase activity (Fig. 3A–C). Interestingly, CHR-6494 treatment significantly increased the ratio of ACD of WT mESCs, similarly to what is observed in Haspin-KO mESCs (Fig. 3D).

To verify whether the ACD ratio change in CHR-6494 treated WT mESCs would lead to similar cell fate defects as it is in the Haspin-KO cells, we conducted differentiation tests including AP staining and EB induction. Indeed, CHR-6494 treatment phenocopied Haspin-KO cells not only in flattened colonies and weaker AP staining (Fig. 3E), but also in significantly reduced EB sizes (Fig. 3F, G).

These data showed that Haspin kinase activity is indispensable to regulates the ACD ratio in mESCs. Moreover, perturbation of ACD with CHR-6494 lead to similar cell fate defects as it is in the Haspin-KO mESCs.

### Haspin influences ACD ratio by regulating the expression of Wnt5a

To look for the downstream factors of Haspin in regulating the ACD ratio in stem cells, we performed RNA-seq analysis of Haspin-KO *vs*. WT mESCs. Compared with WT cells, Haspin-KO cells had 79 upregulated and 79 downregulated differentially expressed genes (DEGs) (Fig. S4A, Table S2). In addition, Gene Ontology (GO) analysis of biological processes showed that the DEGs were involved in translation initiation, stem cell differentiation, telomere maintenance, and skeletal system development (Fig. S4B).

**Fig. 4:**
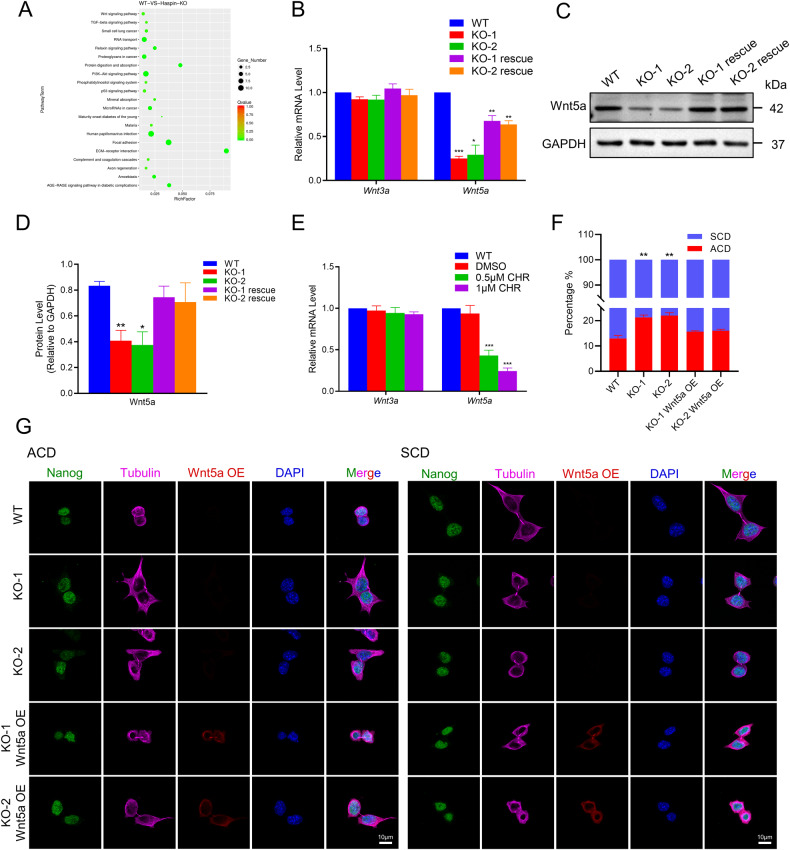
Effect of Wnt5a overexpression in Haspin-KO mESCs on ACD. **A** Kyoto Encyclopedia of Genes and Genomes (KEGG) enrichment analysis of DEGs in Haspin-KO cells compared with WT cells. **B** The relative mRNA expression levels of *Wnt3a* and *Wnt5a* in WT, Haspin-KO, and rescued mESCs (*n* = 3). **C** Western blotting analysis of Wnt5a protein expression in WT, Haspin-KO, and rescued mESCs. **D** The statistical results of the protein expression of (**C**) (*n* = 3). **E** The relative mRNA levels of *Wnt3a* and *Wnt5a* in WT, DMSO, and CHR-6494 treated cells were quantified by qRT‒PCR (*n* = 3). **F** The percentages of ACD and SCD as defined by Nanog fluorescence intensity in WT, Haspin-KO, and Wnt5a-RFP overexpressing mESCs. *n* = 150 from three independent experiments. **G** Representative images of WT, Haspin-KO, and Wnt5a-RFP overexpressing mESCs cultured with N2B27 medium and stained with antibodies against Nanog and Tubulin. Scale bar: 10 μm. The data in (**B**) and (**D**–**F**) are represented as the mean ± SEM. *p < 0.05, **p < 0.01 and ***p < 0.001.

Previous studies have shown that Wnt signaling is associated with ACD [24] and is necessary for the differentiation of mESCs [28]. Notably, Kyoto Encyclopedia of Genes and Genomes (KEGG) pathway analysis also revealed that DEGs enriched in the Wnt signaling pathways were dysregulated in Haspin-KO mESCs (Fig. 4A). Thus, we focused on Wnt signalings for subsequent studies. To experimentally validate if Wnt signaling-related genes are really affected, we selected *Ccnd2*, *Serpinf1,* and *Wnt5a* from the DEG enriched list for qRT‒PCR analysis. And the results were consistent with the RNA-seq data that genes related to the Wnt pathway were significantly downregulated (Fig. S4C).

Wnt signaling is classified into canonical and non-canonical Wnt pathways, represented by Wnt3a and Wnt5a respectively, based on whether this signaling occurs in a β-catenin-dependent or independent manner [29, 30]. Thus, we also measured the expression of *Wnt3a* and *Wnt5a*. Interestingly, Haspin-KO did not affect the expression level of Wnt3a (Fig. 4B). However, *Wnt5a* was significantly reduced in Haspin-KO cells and was rescued by Haspin-eGFP (Fig. 4B–D). Consistently, Wnt5a was also suppressed in WT mESCs treated with Haspin inhibitor CHR-6494 (Fig. 4E). Therefore, we hypothesized that Haspin may regulate ACD in mESCs through Wnt5a. To verify this hypothesis, we expressed Wnt5a-RFP in Haspin-KO mESCs to see if Wnt5a could functionally replenish the absence of Haspin on ACD regulation (Fig. S4D–F). What’s more, overexpression of Wnt5a-RFP in Haspin-KO cells can rescue the flattened cell morphology and EB size (Fig. S4G–I). Strikingly, we found that Wnt5a indeed restored the ACD ratio changes caused by Haspin-KO (Fig. 4F, G), suggesting Haspin influences ACD ratio via Wnt5a.

### Wnt5a knock down phenocopied Haspin-KO with increased ratio of ACD and differentiation defects in mESCs

**Fig. 5:**
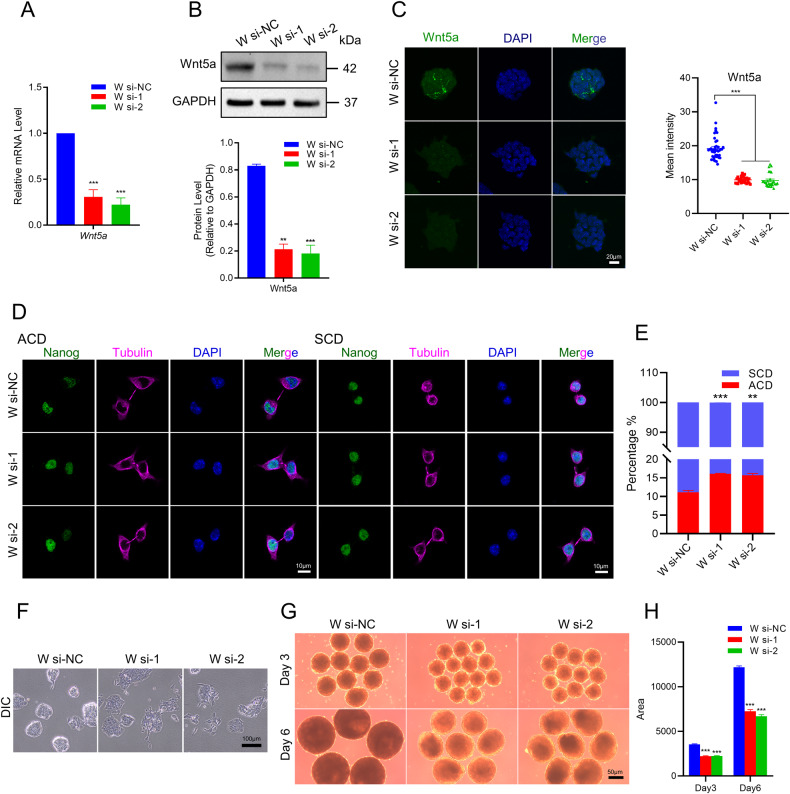
Effect of Wnt5a on developmental potencies. **A** Relative mRNA levels of *Wnt5a* in cells transfected with Wnt5a RNAi cells (W si-NC, W si-1, W si-2) by qRT‒PCR (*n* = 3). **B** The protein levels of Wnt5a in Wnt5a RNAi cells. The statistical results of the protein level of (**B**) (*n* = 3). **C** Immunofluorescence and statistical analysis of cells transfected with Wnt5a in W si-NC, W si-1, and W si-2. Scale bar: 20 μm. **D** Representative images of mESCs transfected with Wnt5a si-NC, si-1 and si-2 cultured with N2B27 medium and stained with antibodies against Nanog and Tubulin. Scale bar: 10 μm. **E** The ratio of ACD and SCD defined by Nanog fluorescence intensity in mESCs transfected with W si-NC, W si-1, and W si-2. *n* = 150 from three independent experiments. **F** Phase-contrast microscopy images of mESCs transfected with W si-NC, W si-1, and W si-2 mESCs. Scale bar: 100 μm. **G** Representative images of EB were obtained from cells transfected with Wnt5a RNAi on Day 3 and Day 6. Scale bar: 50 μm. **H** The statistics analysis of EB area on Day 3 and Day 6. *n* = 20 for Day 3 and Day 6. The data in (**A**–**C**), (**E**), and (**H**) are represented as the mean ± SEM. *p < 0.05, **p < 0.01 and ***p < 0.001.

Wnt signaling pathway is reported to be involved in ACD for the differentiation of mESCs [24, 28]. For example, Wnt3a-coated beads can orient the division plane of mESCs to achieve ACDs [24]. However, the effect of Wnt5a on ACD in stem cells remains elusive. To further explore if Wnt5a is directly involved in regulating ACD in mESCs, we knocked down Wnt5a by siRNA. Efficient knockdown of Wnt5a was verified by qRT‒PCR, western blotting, and immunofluorescence (Fig. 5A–C). Next, we quantified the ACD ratio of these cells, finding that Wnt5a KD could significantly increase the ratio of ACD (Fig. 5D, E), which is consistent with our observations in Haspin-KO mESCs.

Moreover, subsequent differentiation tests in Wnt5a-KD mESCs showed that lack of Wnt5a resulted in flattened cell morphology with distinct early differentiation (Fig. 5F) and significantly reduced EB sizes (Fig. 5G, H). In summary, our data suggested that Wnt5a was important for ACD and developmental potential in mESCs.

### Haspin regulates Wnt5a expression through Pax2

**Fig. 6:**
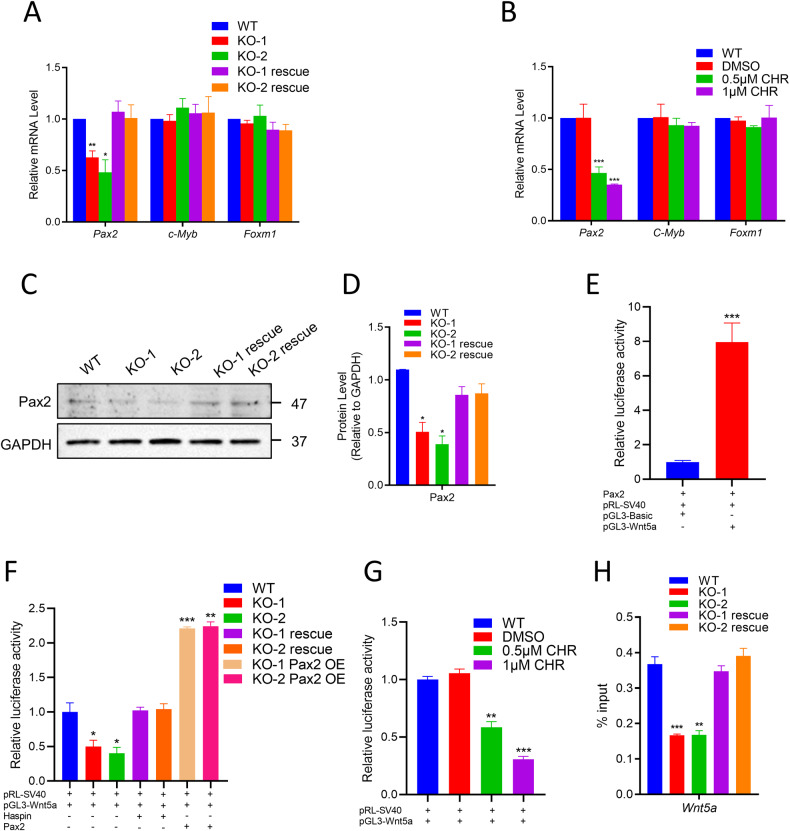
Wnt5a is regulated by Haspin through Pax2 in mESCs. **A** Relative mRNA expression of the *Wnt5a* transcriptional activators *Pax2*, *c-Myb,* and *Foxm1* in WT, Haspin-KO, and rescued cells (*n* = 3). **B** mRNA levels of *Pax2*, *c-Myb* and *Foxm1* in WT, DMSO, 0.5 μM CHR-6494 and 1 μM CHR-6494 treated cells (*n* = 3). **C** Protein levels of Pax2 in WT, Haspin-KO, and rescued cells. **D** The statistical results of Pax2 protein expression (*n* = 3). **E** Pax2-CDS, pRL-SV40, pGL3-Basic, or pGL3-Wnt5a promoter were co-transfected into WT E14 cells. After 36 h, the luciferase activity of Fluc and Rluc was measured, and Fluc/Rluc was calculated. **F** pRL-SV40 and the pGL3-Wnt5a promoter were co-transfected into WT, Haspin-KO, rescued cells, and Pax2 OE cells, respectively. **G** pRL-SV40 and the pGL3-Wnt5a promoter were co-transfected into WT cells. After 8 h, the transfected cells were changed to medium containing WT, DMSO, 0.5 μM CHR-6494, and 1 μM CHR-6494 and incubated for 24 h. **H** ChIP‒qPCR analysis of Pax2 binding to in the Wnt5a promoter region in WT, Haspin-KO, and rescue cells. IgG was used as a negative control. The data in (**A**), (**B**), and (**D**–**H**) are represented as the mean ± SEM. *p < 0.05, **p < 0.01, and ***p < 0.001.

To investigate how Haspin regulates the level of Wnt5a, we used qRT‒PCR to measure the expression of the reported transcriptional activator of Wnt5a (*Pax2* [31]*, c-Myb* [32], *Foxm1* [33]). Interestingly, only *Pax2* was markedly reduced in the Haspin-KO mESCs (Fig. 6A). We also examined the effect of the Haspin inhibitor CHR-6494 on the expression of Wnt5a transcriptional activators. Consistent with the results in the Haspin-KO cells, Pax2 was also reduced in CHR-6494 treated WT mESCs (Fig. 6B). Then, we also examined the protein level of Pax2 and found that Pax2 was reduced in Haspin-KO cells but elevated after Haspin-eGFP overexpression (Fig. 6C, D). To validate that *Pax2* was transcriptional responsive to *Wnt5a* in mESCs, we performed luciferase reporter assay with *Wnt5a* promoter and *Pax2*. Indeed, Pax2 enhanced the expression of pGL-3 *Wnt5a* promoter vector luciferase, compared to the pGL-3 basic vector control (Fig. 6E).

To further understand the regulatory relationship among Haspin, Pax2, and Wnt5a, we co-transfected pRL-SV40 and pGL3-*Wnt5a* promoter into WT, Haspin-KO, Haspin-eGFP rescue, and Haspin-KO + Pax2 OE cells respectively. We found that the luciferase activity of the pGL3-*Wnt5a* promoter was reduced in Haspin-KO cells and recovered in both Haspin-eGFP rescue and Haspin-KO + Pax2 OE cells (Fig. 6F). Similarly, CHR-6494 treatment also reduced pGL-3 *Wnt5a* promoter activity in WT mESCs (Fig. 6G). Moreover, we analyze the binding ability between *Pax2* and the *Wnt5a* promoter in Haspin-KO cells by ChIP‒qPCR. Haspin-KO significantly reduced the binding of Pax2 to the *Wnt5a* promoter (Fig. 6H). Taken together, these data suggest that Pax2 works as a transcriptional activator of Wnt5a and its expression is regulated by Haspin in mESCs. Deficiency of Haspin reduced the expression of Wnt5a by decreasing the expression of Pax2 and the binding ability of Pax2 to *Wnt5a* promoter, then further affects ACD and cell fate in mESCs.

## Discussion

**Fig. 7:**
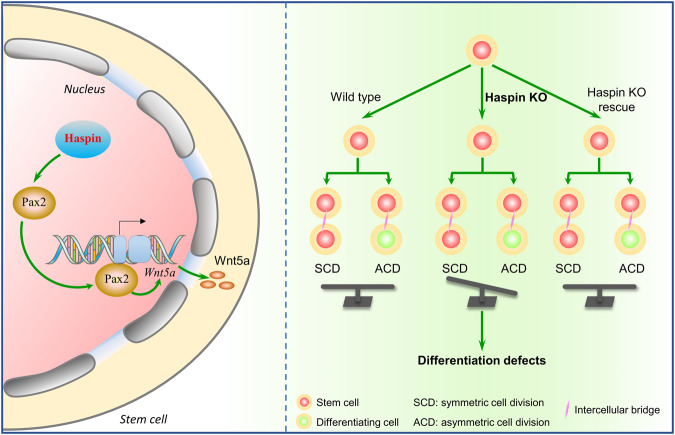
Haspin balances the ratio of ACD through Wnt5a and regulates cell fate decisions in mESCs. In mESCs, Haspin regulates the expression of Wnt5a through Pax2, further balances the ratio of ACD and regulates cell fate decision. Deficiency of Haspin caused an increased ratio of ACD and cumulatively regulates cell fate decisions, which all can be rescued by the replenish of Haspin-eGFP.

Here, we report that Haspin is indispensable for mESCs to maintain a balanced ratio of ACD, which is essential for normal development and homeostasis. Both Haspin-KO and Haspin-specific inhibitor treatment caused similar phenotype of ACD ratio changes, which cumulatively regulates cell fate decisions. To look for the downstream candidates, we performed RNA-seq and found that Wnt5a was significantly downregulated in Haspin-KO cells. Moreover, knockdown of Wnt5a by RNAi phenocopied Haspin-KO cells in both ACD ratio shift and lineage development defects, while overexpression of Wnt5a in Haspin-KO cells restored the ACD ratio. Furthermore, we found that decreased Wnt5a signaling occurs due to the lack of its upper stream transcriptional activator, Pax2, in Haspin-KO cells, which is responsible for ACD ratio disproportion and cumulative regulates cell fate decisions. Taken together, our results showed that Haspin balances the ACD ratio through Wnt5a and regulates cell fate decision in mESCs (Fig. 7).

Haspin-mediated H3T3ph was reported to be essential for the asymmetric separation of old *vs*. new histones enriched chromosomes in *Drosophila* male GSCs. Perturbation of H3T3ph leads to stem cell loss or germline tumors due to changes in the ACD patterns [7]. These results suggest that Haspin is involved in regulating ACD. Consistently, our data show that both Haspin-KO and Haspin-specific inhibitor treatment significantly increased the ACD ratio and led to cumulative regulates cell fate decisions.

Further investigation by RNA-seq of Haspin-KO mESCs revealed that Wnt signaling components were significantly affected. The Wnt signaling pathway plays a crucial role in gene transcription, stem cell fate decisions and tumorigenesis [34]. Previous studies have demonstrated that Wnt signaling is essential for regulating ACD and further cell fate in different species from metazoans to vertebrates [35–40], including mESCs [24, 26, 41]. For example, Wnt signaling regulates the ACD of ectodermal cells to promote ciliated cell differentiation in *Xenopus* [37]. Wnt5a, a glycoprotein, is a representative of the non-canonical pathway and has been reported to be essential for ESC differentiation into endothelia and vascularization [28], endometrium-like cells [42], and osteogenic lineage decisions [43]. It is possible that ACD plays a vital role in the phenotypes observed in these studies. Interestingly, Wnt3a-coated beads can orient the division plane of mESCs to achieve ACDs [24]. However, the endogenous effect of Wnt5a on ACD in stem cells remains elusive. Our data showed that overexpression of Wnt5a in Haspin-KO mESCs can restore the defects caused by the lack of Haspin, especially the ACD ratio changes, indicating that Haspin balances ACD ratio through Wnt5a in mESCs.

Moreover, our data showed both *Pax2* and *Wnt5a* were transcriptionally disrupted in Haspin-KO cells. Though histone acetylation and methylation have been intensely studied for their important roles in transcriptional regulation, it is clear that histone phosphorylation also influences gene transcription. Indeed, phosphorylation of H3 are involved in regulation of a variety of genes in response to growth factors, steroid hormones, and cell stress [44–47]. Our results showed that mRNA level of *Pax2*, a transcriptional activator of *Wnt5a*, was significantly decreased in Haspin-KO cells, suggesting Haspin influences Wnt5a expression through Pax2 in mESCs. However, it is still unknown how Haspin regulates Pax2 at transcriptional level. One possibility is that H3T3ph may affect the trimethylation of histone H3 at lysine 4 (H3K4me3) due to spatial proximity between these two residues [48–50]. As we all know, H3K4me3 is a pivotal epigenetic modification for active gene transcription [51, 52]. Indeed, we found that Haspin could affect the level of H3K4me3 (data not shown). However, this hypothesis needs to be further verified in future works.

In summary, our findings revealed that Haspin balances the ratio of asymmetric cell division through Wnt5a and regulates cell fate decisions in mESCs.

## Methods

### Cell culture

E14 mESCs were purchased from the Chinese Academy of Sciences Stem Cell Bank. They were cultured at 37 °C in 5% CO_2_ in an incubator. The culture medium was Knockout DMEM (Gibco, 10829-018), added with 15% fetal bovine serum (FBS, Gibco, 10099-141), 1× MEM nonessential amino acids (Gibco, 11140-050), 1× GlutaMAX (Gibco, 35050-061), 50 μM 2-Mercaptoethanol (Sigma, M3148), 1000 IU/mL LIF (Millipore, ESG1107), 1 μM PD0325901 (Selleck, S1036) and 3 μM CHIR99021 (Selleck, S2924) [25]. mESCs were maintained on mitomycin C-treated mouse embryonic fibroblasts (MEFs) for passage or in 0.1% gelatin-coated dishes for assays. The medium was changed every day, and the cells were passaged every 3 days.

### Haspin knockout in mESCs

To knockout Haspin, we designed two guide RNAs (gRNAs) targeting Haspin coding sequences. The gRNAs were annealed and then cloned into the BbsI-digested vector pX459. These two Haspin gRNA plasmids were co-transfected into WT E14 cells using Lipofectamine 2000 (Lip2000, Invitrogen, 11668019). 24 h after transfection, the medium was changed to ES medium supplemented with 1.5 μg/mL puromycin for 3 days, and the surviving single clones were picked and transferred to 96-well plates. To obtain Haspin-KO cells, we used immunostaining of H3T3ph to select H3T3ph negative single clones. Then, the genomic DNA fragments of the Haspin-KO cells were amplified by PCR and sequenced to confirm that the Haspin gene was disrupted.

### Induction of asymmetric stem cell division

When assaying asymmetric stem cell division ability, E14 mESCs were switched to N2B27 medium and incubated for 16 h on gelatin-coated glass-bottom cell culture dishes (Nest, 801001) after passage. N2B27 medium includes DMEM/F12 (Gibco, 11320-033), Neurobasal (Gibco, 21103-049) (1:1), 50 μM 2-mercaptoethanol, 0.5% N2 (Gibco, 17502-048), 1% B27 (Gibco, 17504-044), 0.033% BSA 7.5% solution (Thermo Fisher, 15260037) and 1× GlutaMAX (Gibco, 35050-061) [24]. After 16 h, immunofluorescence staining experiments were immediately performed on the cells, and then the cells were prepared for confocal imaging. *Nanog*, one of the core pluripotency markers, has always been used to determine ACD ratio [24–26]. When the signal ratio of Nanog between the two daughter cells is greater than 1.2, it is considered as ACD [53].

### Plasmid construction and overexpression

The Chek2-eGFP and Chek2-RFP vectors were obtained from Dr. Zhiyong Mao. We amplified the coding sequences of Haspin and Wnt5a from cDNA of mESCs and then subcloned them into the Chek2-eGFP vector to displace the Chek2 coding sequence. For overexpression, Haspin-eGFP or Wnt5a-RFP vector was transfected into E14 mESCs using Lip2000. After 3 days of transfection, GFP-positive cells were purified with flow cytometry using green fluorescence. The efficiency of overexpression was measured by qRT‒PCR and western blotting.

### siRNAs

E14 cells were transfected with siRNA targeting *Haspin* or *Wnt5a* for 48 h using Lip2000 in accordance with the manufacturer’s instructions. The *Haspin* and *Wnt5a* siRNAs were synthesized by Ribobio (Guangzhou, China), and their target DNA sequences are shown in Supplemental Table 1.

### Haspin kinase inhibitor CHR-6494

The Haspin kinase inhibitor CHR-6494 (MCE, HY-15217) was dissolved in DMSO to generate a 10 mM stock solution and then stored at −20 °C [27]. After cell passage and adherence, the medium was changed to 0.5 μM or 1 μM CHR-6494 added ES medium or N2B27 medium, and 1 μM DMSO was used as a control. The medium was changed every day.

### Cell synchronization

To measure the level of H3T3ph, we synchronized the mESCs. Cells were synchronized to metaphase through treatment with 1 μg/mL colchicine (Sigma, C3915) for 8 h, and then, the cells were collected for western blotting or immunofluorescence staining.

### Alkaline phosphatase staining

mESC alkaline phosphatase (AP) staining was performed according to the manufacturers’ instructions using the Alkaline Phosphatase Detection Kit (Millipore, SCR004). First, the cells were fixed for 1 min at room temperature with 4% paraformaldehyde in PBS and then washed for 5 min with 1× PBST. Next, Fast Red Violet solution and Napthol AS-BI phosphate solution were applied to the mESCs for 10 min in the dark. Stained cells were washed with 1× PBS and examined under an inverted microscope.

### Growth curves

Approximately 20,000 cells were seeded into each well of 24-well plates (Thermo, 142475). Then, growth curves were generated at 24, 48, 72, 96, and 120 h, and three wells of each line were counted to determine the cell number. Cell number was measured via Countess II (Thermo) according to the manufacturer’s protocol. The remaining cells were washed with 1× PBS, and the medium was replaced daily with fresh medium.

### Cell cycle analysis

E14 mESCs were plated in a six-well cell culture plate (Thermo, 140675). Three days later, the cells were trypsinized and fixed in cold 70% ethanol overnight at 4 °C. The cells were resuspended and incubated with RNaseA solution at 37 °C for 30 min, followed by incubation with 50 μg/mL of propylidine iodide (PI) solution for another 30 min at room temperature. Then, BD FACS Aria ll was used to assess the frequency of cells in the G1 phase, S phase, and G2/M phase.

### Embryoid body generation

The hanging drop method was used to generate embryoid bodies (EBs). E14 cells were dissociated and resuspended in EB medium at a concentration of 1–1.5 × 10^5^ cells/mL. EB media containing Knockout DMEM, 15% FBS, 1× MEM nonessential amino acids, 1× Sodium Pyruvate, and 0.1 mM 2-Mercaptoethanol [54]. Then, 20 μL hanging drops were cultured for three days in the lid of a 10 cm dish. To prevent the hanging drop from drying out, 1× PBS was placed at the bottom of the lid. After three days, EBs were collected, transferred into ultralow adhesion plates (Corning, 3474) and cultured in EB media for another 3 days. EB samples were collected and then subjected to qRT‒PCR to analyze the gene expression levels.

### Teratoma formation

Mice were housed and treated according to the Animal Research Institute Committee guidelines of Tongji University, Shanghai, China. For teratoma formation, E14 cells were trypsinized to produce single cells. For each cell type, a total of 1 × 10^6^ cells were resuspended in 100 μL EB medium and subcutaneously injected into the two flanks of immunodeficient NOD-SCID 8-week-old male mice (*n* = 4 tumors/group). Four weeks after inoculation, the teratomas were harvested and weighed. Then, these teratomas were collected in 4% PFA, embedded in paraffin, and sectioned in a microtome. Sections of each teratoma were stained with hematoxylin/eosin (H&E) and further analyzed.

### Quantitative real-time PCR (qRT‒PCR)

Total RNA was extracted from WT and Haspin-KO mESCs, EBs and teratomas using TRIzol (Invitrogen, 10296010) according to the protocol. Total RNA was quantified using a NanoDrop 2000 spectrophotometer (Thermo). Then, reverse transcription into cDNA was performed by the PrimeScript RT Reagent Kit with gDNA Eraser (Takara, RR047). qRT‒PCR was performed using TB Green Fast qPCR Mix (Takara, RR430) with a reaction volume of 20 μL and the Bio-Rad CFX Connect system (Bio-Rad). The relative mRNA expression levels were normalized to *GAPDH* and analyzed using the 2-delta-delta-Ct method to calculate the relative fold change. Primer sequences are listed in Supplementary Table 1.

### Immunofluorescence staining

For immunofluorescence staining, cells grown on glass-bottom cell culture dishes were fixed with 4% paraformaldehyde at room temperature for 30 min. The samples were permeabilized with PBST (0.2% Triton X-100) at room temperature for 30 min. Then, the cells were blocked with 5% BSA at room temperature for 1 h. Then, the samples were incubated overnight with primary antibodies at 4 °C. Furthermore, the samples were washed three times with PBST and incubated at room temperature for 1 h with secondary antibodies and Hoechst 33342 (Invitrogen, H3570). Then, the samples were washed in PBST three times at room temperature and stored in the dark. The images were captured using a Zeiss-LSM880 inverted microscope. Data were processed using Zeiss software and ImageJ software.

### Western blotting

For protein extraction, mESCs were lysed using RIPA buffer (Beyotime, P0013B) supplemented with protease inhibitors (Beyotime, P1005) and phosphatase inhibitors (Beyotime, P1081), and then, the lysates were centrifuged at 12,000 rcf and 4 °C for 20 min. The protein concentration of the supernatant was determined using the BCA Protein Assay (Thermo, 23227) and then normalized. Equal amounts of protein samples were separated using 15% SDS‒PAGE gels and then transferred to 0.2 μm PVDF membranes (Millipore, ISEQ00010). Membranes were blocked with TBST supplemented with 5% BSA at room temperature for 1 h and then incubated with primary antibodies at 4 °C overnight. Next, the membranes were washed three times using TBST followed by HRP-conjugated secondary antibodies for 1 h at room temperature. The membranes were visualized with the chemiluminescence reagent ECL (Thermo, 34577).

### RNA-seq and data analysis

RNA was extracted from Haspin-KO and control mESCs using TRIzol. Then, 1 μg RNA was prepared to generate a sequencing library. Libraries were sequenced on the IIIumina NovaSeq 6000 (Berry genomics) according to the protocol. For data analysis, the trimmed reads (using Cutadapt, v1.9.1) were mapped to the mouse genome (mm10) using Hisat2 (v2.0.1). Differentially expressed gene (DEG) analysis was performed using DESeq2. The significant DEGs were identified with *P* values < 0.05 and fold change >2, and then GOSeq (v1.34.1) was used to identify Gene Ontology (GO) terms.

### Dual-luciferase reporter assay

We amplified fragments of the Wnt5a promoter (~2 kb upstream of the TSS) and Pax2 CDS, and then, we cloned Wnt5a into the pGL3 luciferase reporter vector, and Pax2 CDS into the CAG-eGFP overexpression vector. mESCs were seeded in 24-well plates at a density of 2 × 10^5^ cells/mL. The next day, Pax2-eGFP, pRL-SV40, pGL3-Basic, or pGL3-Wnt5a promoter were co-transfected into cells using Lip2000 and cultured for 36 h. On the other hand, pRL-SV40 and pGL3-Wnt5a promoter were co-transfected into WT cells. After 8 h, the transfected cells were administered medium to containing WT, DMSO, 0.5 μM CHR-6494, and 1 μM CHR-6494 and incubated for 36 h. Then, the cells were harvested and lysed to measure the luciferase activity according to the manufacturer’s protocol (Promega, E1910).

### Chromatin immunoprecipitation

Chromatin immunoprecipitation (ChIP) assays were performed by the Magna ChIP A/G kit (Millipore) according to the protocol. Briefly, mESCs were cross-linked and then sonicated into 200–500 bp fragments, which were next immunoprecipitated with antibodies against Pax2 (Cell Signaling Technology, 9666) or IgG. Then precipitated chromatin DNA was then recovered and analyzed by qRT‒PCR assays.

### Antibodies

The following primary antibodies were used for western blotting or immunofluorescence staining: anti-H3T3ph (Abcam, ab130940), anti-H3S10ph (Abcam, ab14955), anti-GAPDH (Proteintech, HRP-60004), anti-caspase-3 (Cell Signaling Technology, 9662S), anti-cleaved caspase-3 (Cell Signaling Technology, 9664S), anti-α-tubulin (Active motif, 39527), anti-Nanog (Abcam, ab80892), anti-Sox2 (Abcam, ab79351), anti-Oct4 (Abcam, ab27985), anti-Klf4 (Abcam, ab129473), anti-Wnt5a (Abcam, ab235966), and anti-Pax2 (Cell Signaling Technology, 9666).

### Statistical analysis

All the statistical data are presented as the mean ± SEM. Each test was repeated at least three times, and the results were analyzed by SPSS 20.0. Statistical significance between groups was determined using unpaired two-tailed Student’s t tests. *P < 0.05, **P < 0.01 and ***P < 0.001 were considered statistically significant differences, and *P* > 0.05 was considered not significant.

### Supplementary information

Figure S1

Figure S2

Figure S3

Figure S4

Supplementary Figure Legends

Original Data File

Supplemental Table S1

Supplemental Table S2

## Data Availability

The accession number of RNA-seq data reported in this paper is GEO: GSE220824. The source data presented in this study are available from the corresponding author upon request.
