# Supplementary figures and images for: Haspin balances the ratio of asymmetric cell division through Wnt5a and regulates cell fate decisions in mouse embryonic stem cells
Source: Cell Death Discov. 2023 Aug 23;9:307. doi: 10.1038/s41420-023-01604-w (PMC10447528; doi:10.1038/s41420-023-01604-w)

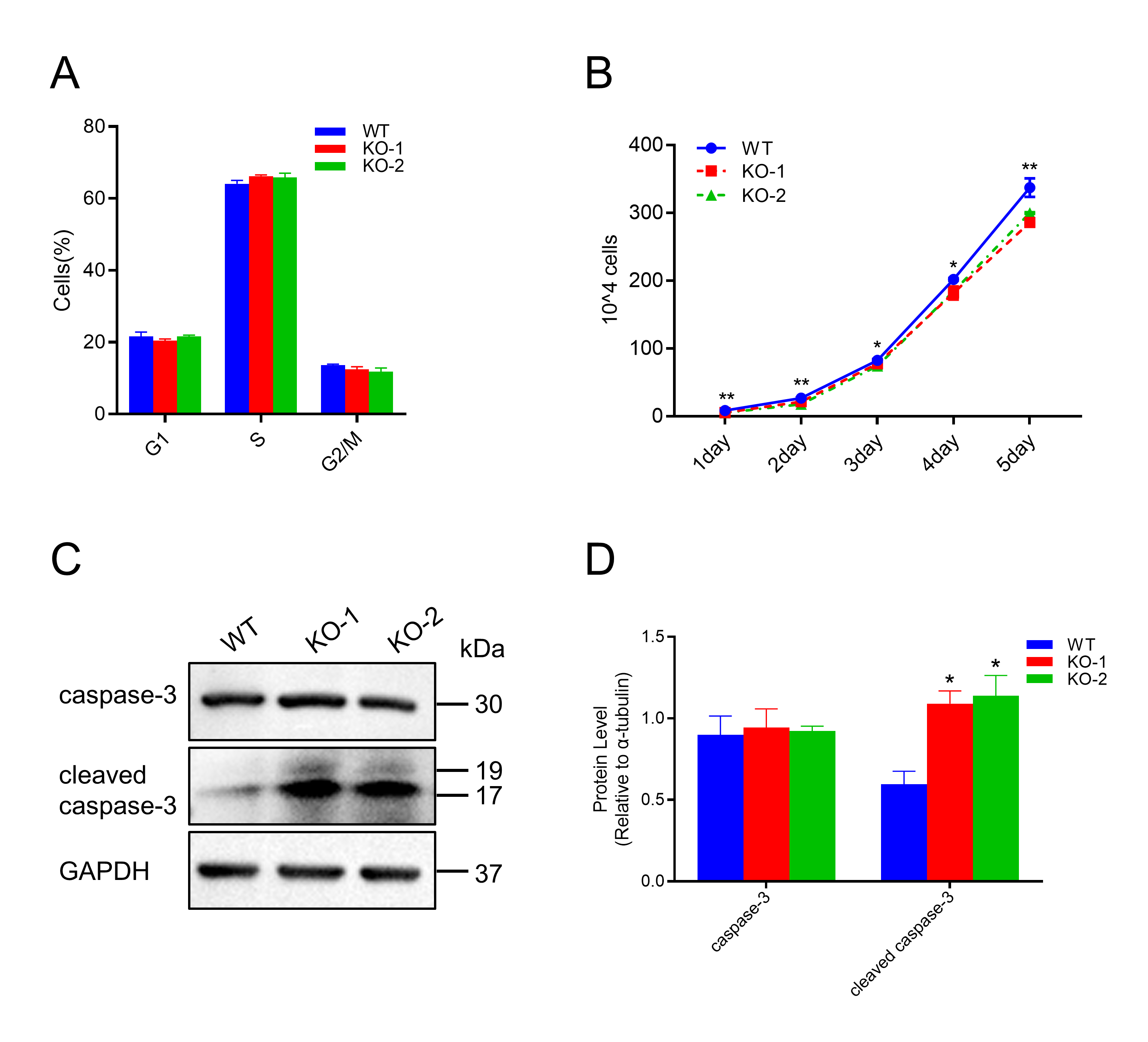

Supplement: Supplementary file 1 — Figure S1 [file 41420_2023_1604_MOESM1_ESM.tif]

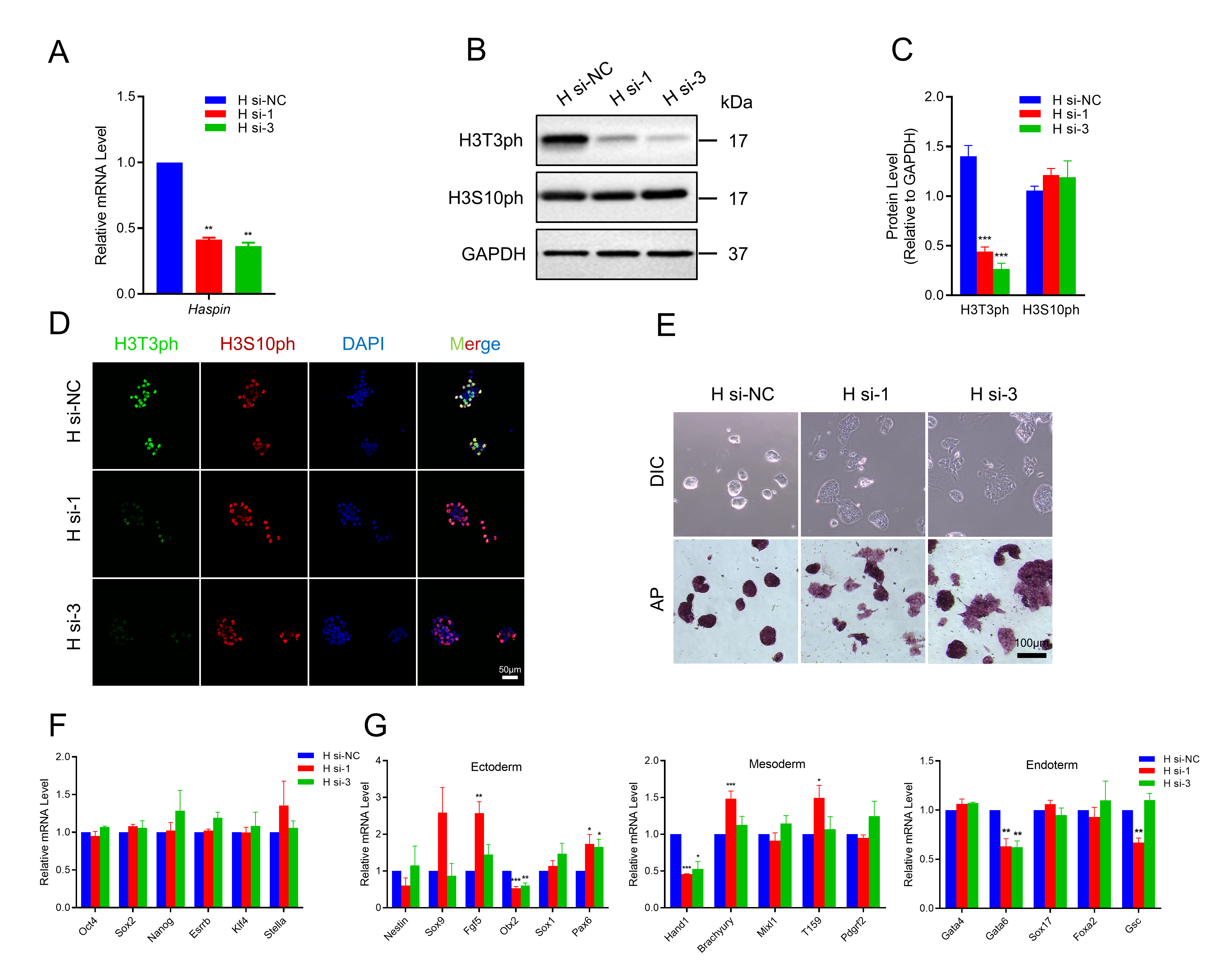

Supplement: Supplementary file 2 — Figure S2 [file 41420_2023_1604_MOESM2_ESM.tif]

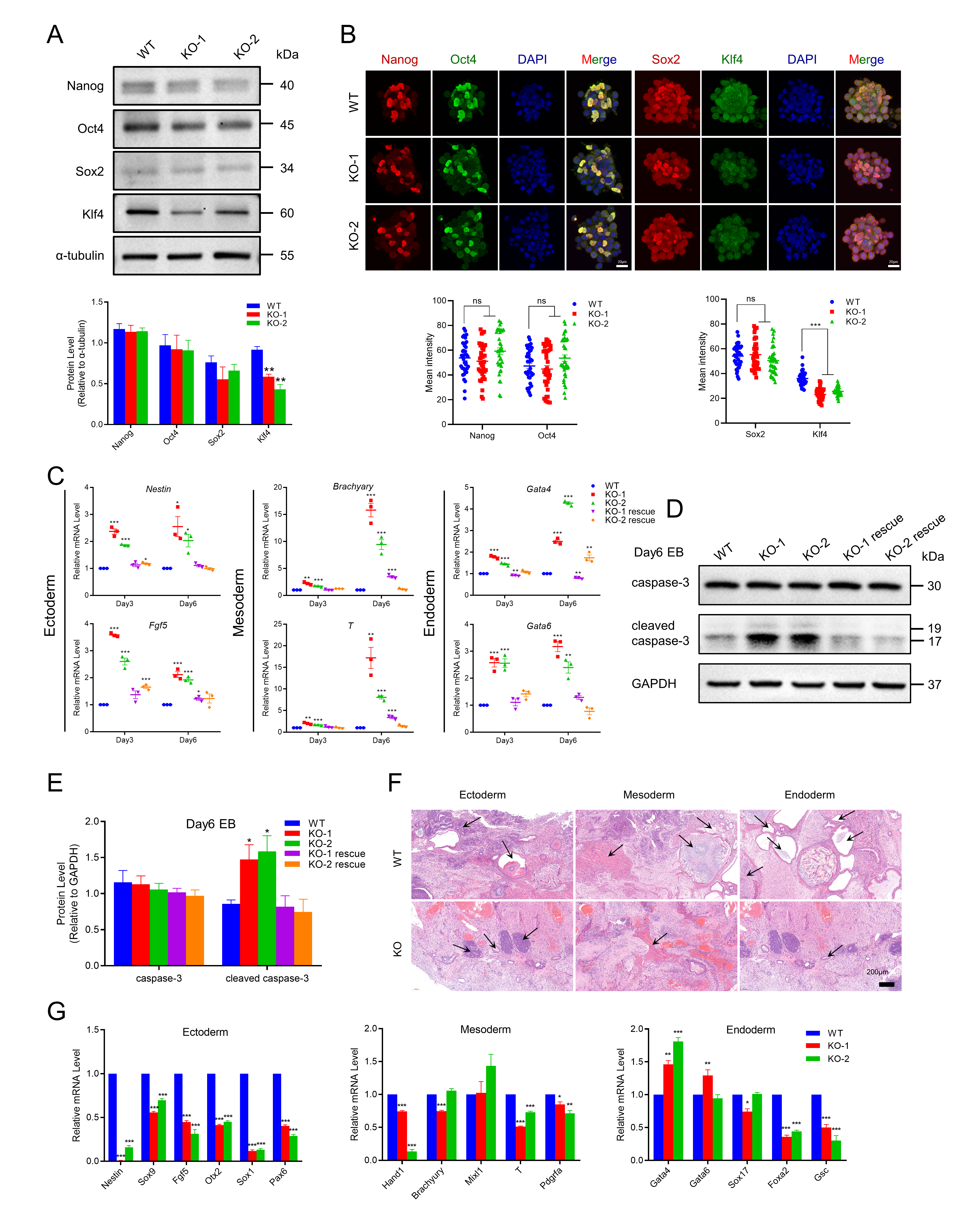

Supplement: Supplementary file 3 — Figure S3 [file 41420_2023_1604_MOESM3_ESM.tif]

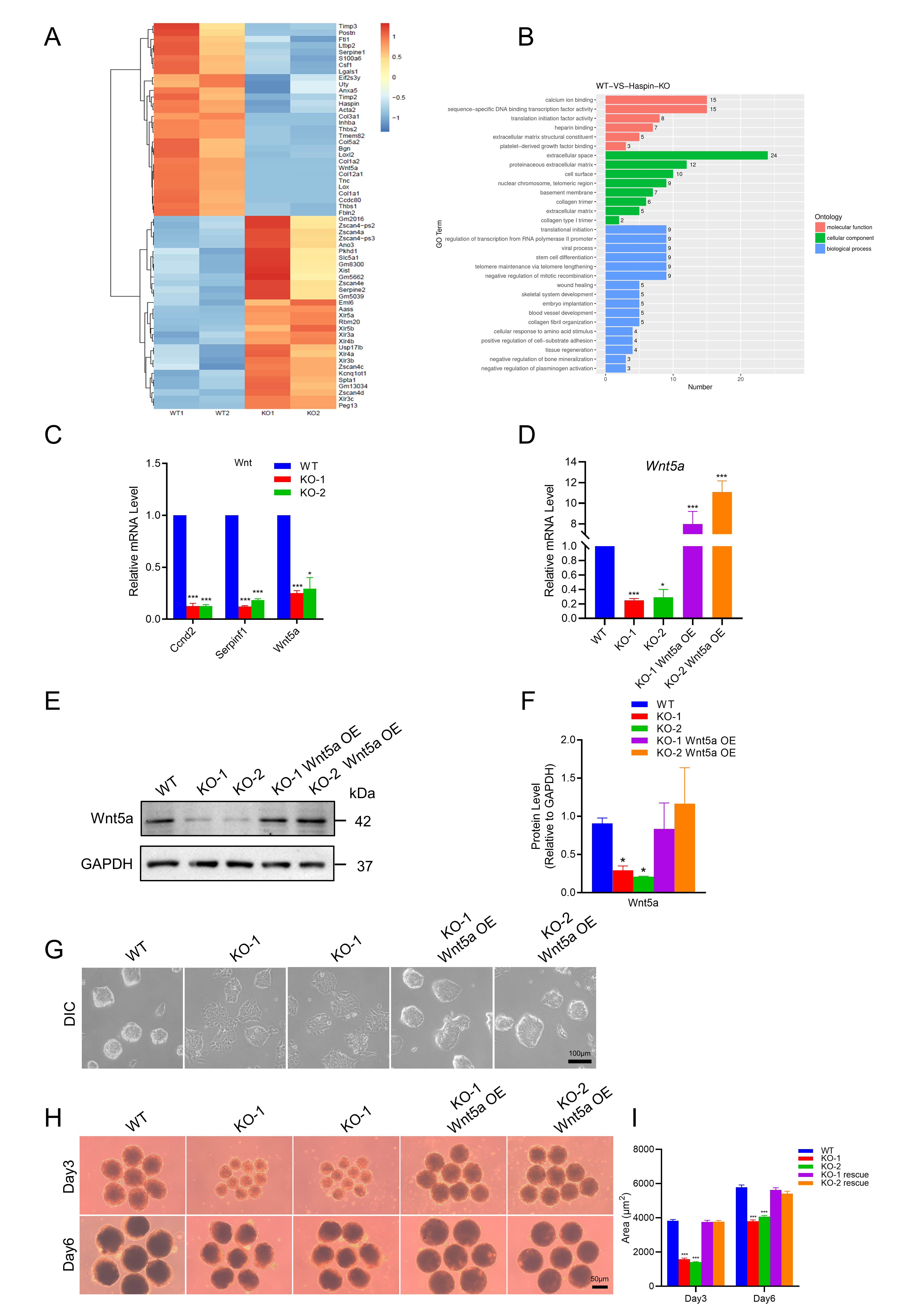

Supplement: Supplementary file 4 — Figure S4 [file 41420_2023_1604_MOESM4_ESM.tif]

Figure 1D

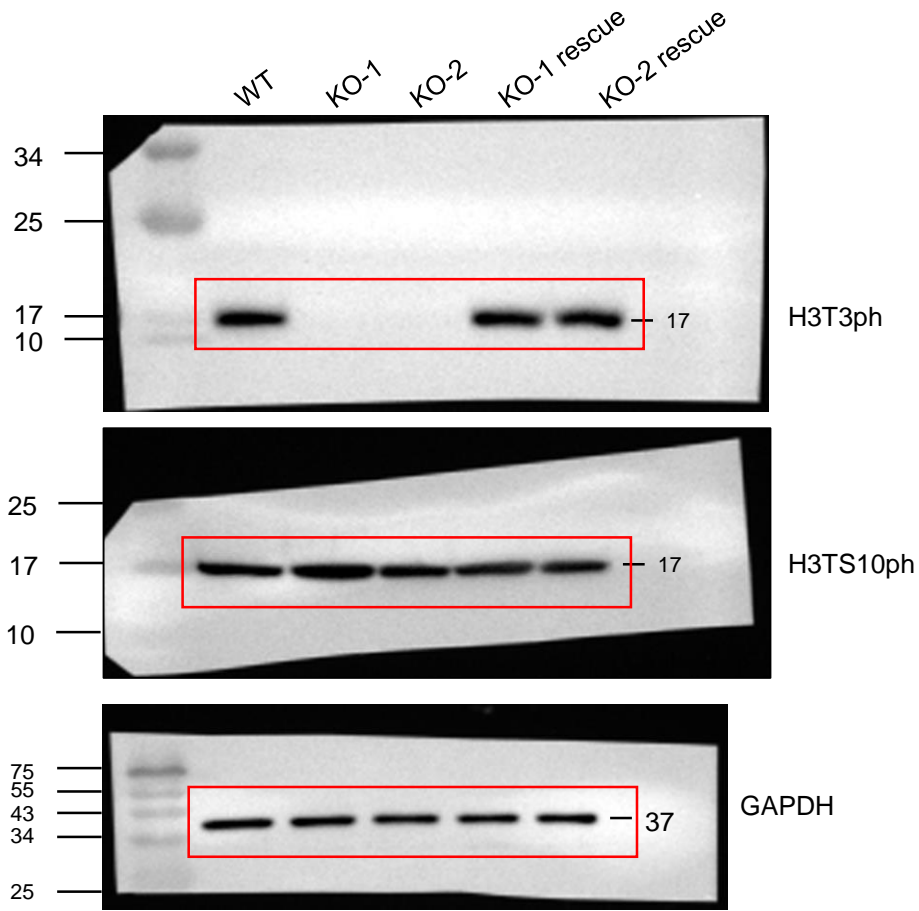

Figure 3A

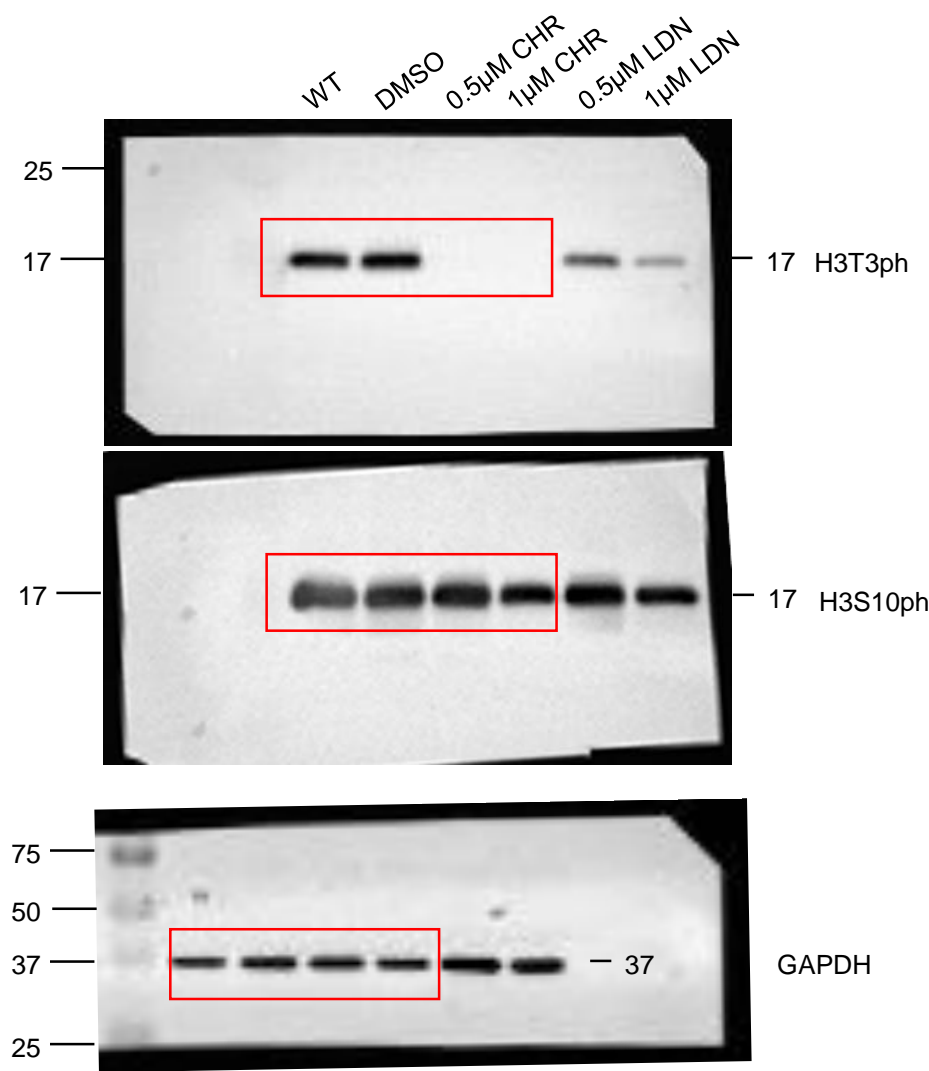

**Figure 4C**

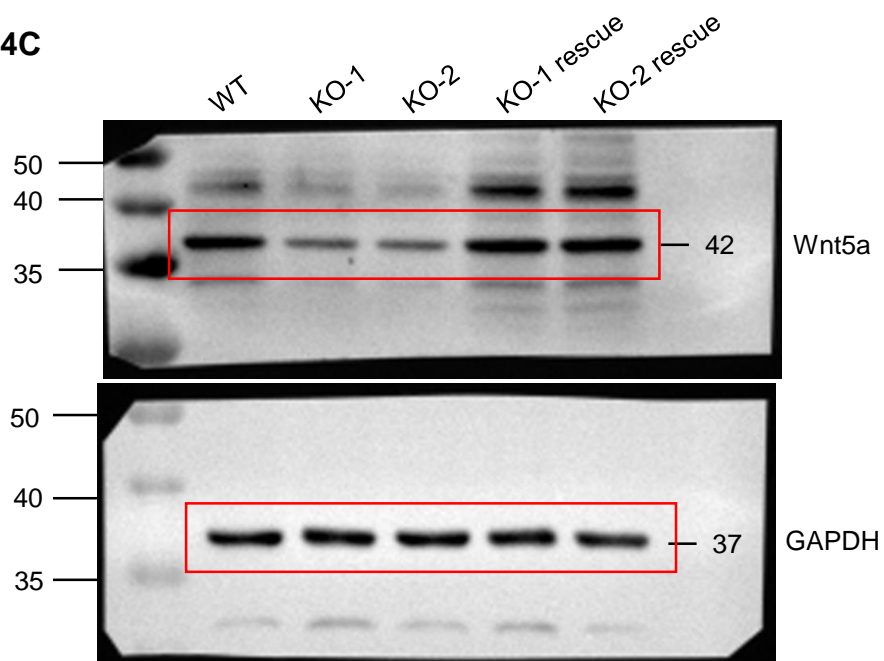

**Figure 5B**

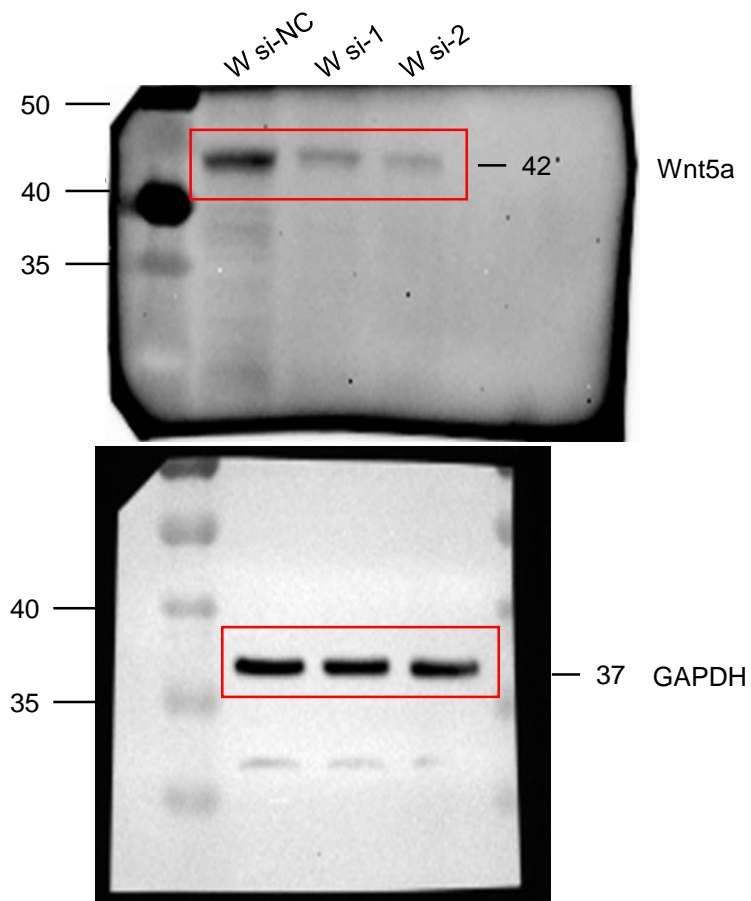

**Figure 6C**

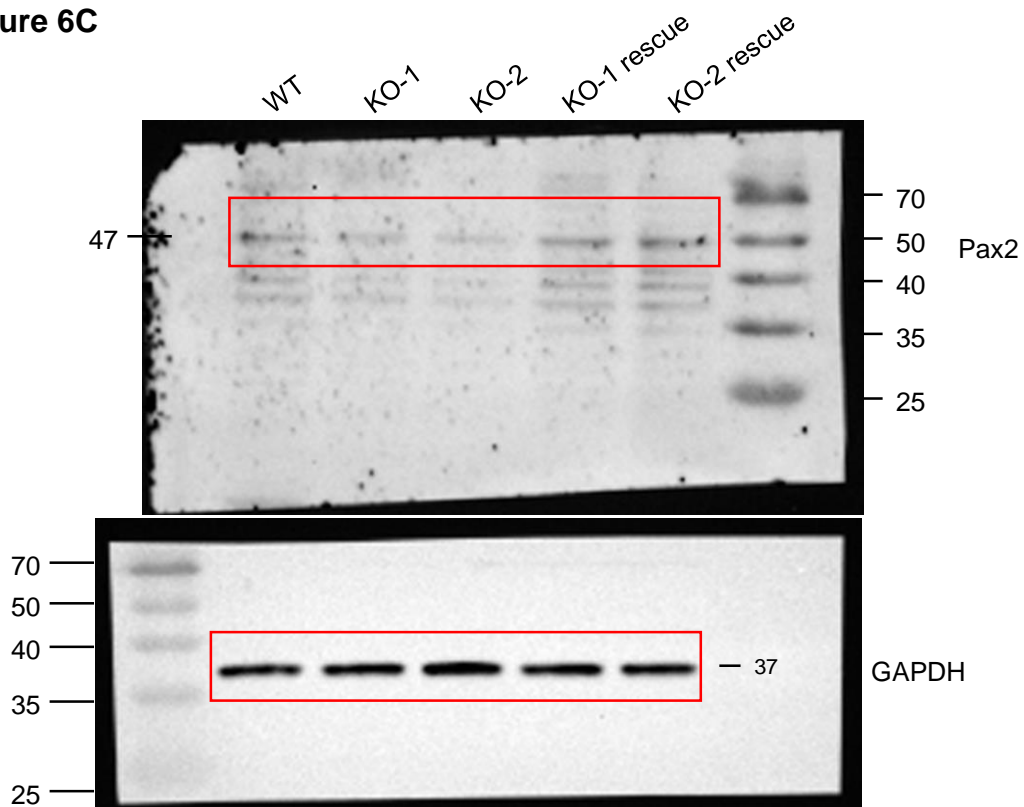

**Figure S1C**

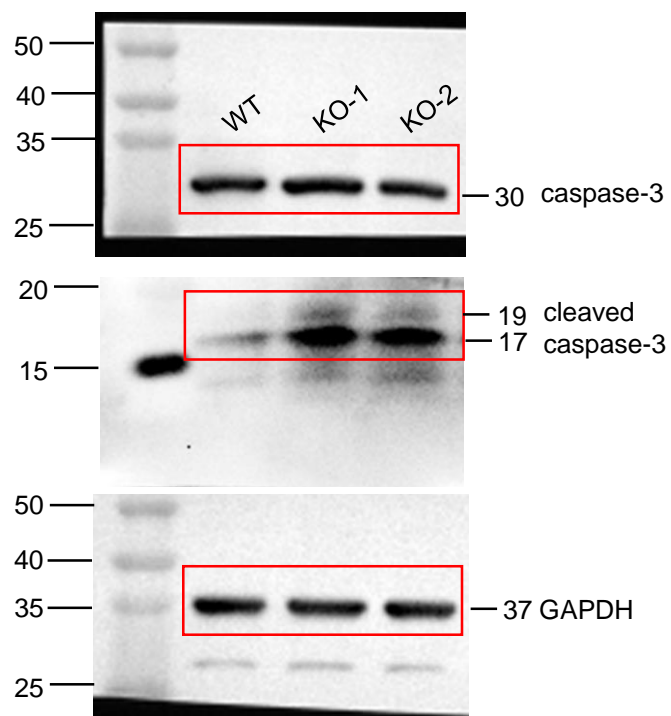

**Figure S2B**

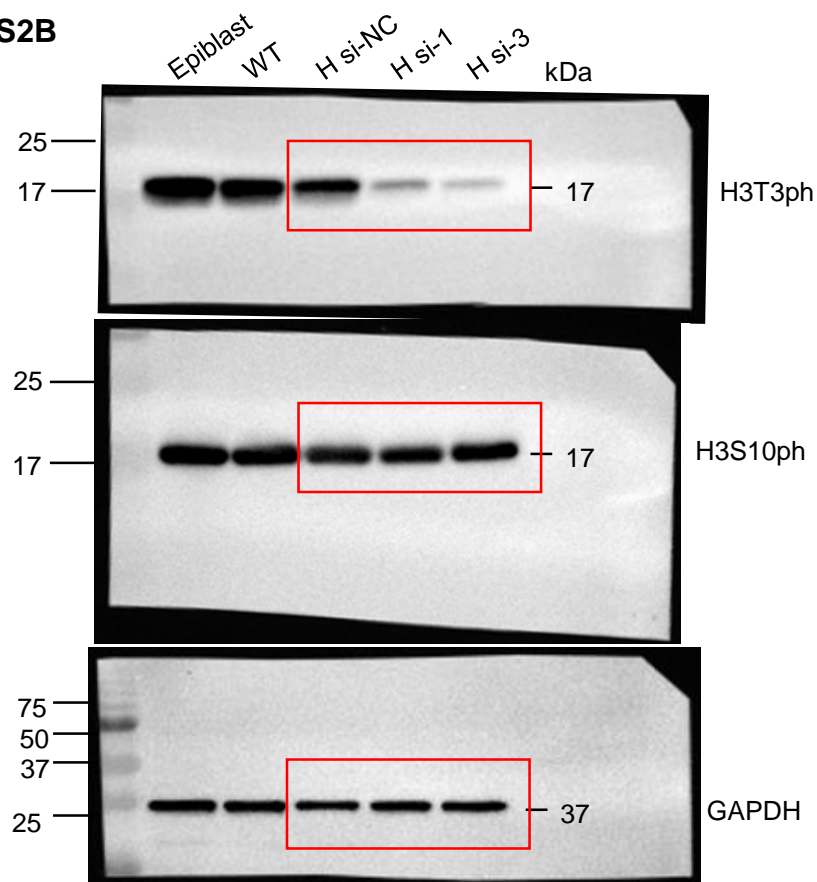

Figure S3A

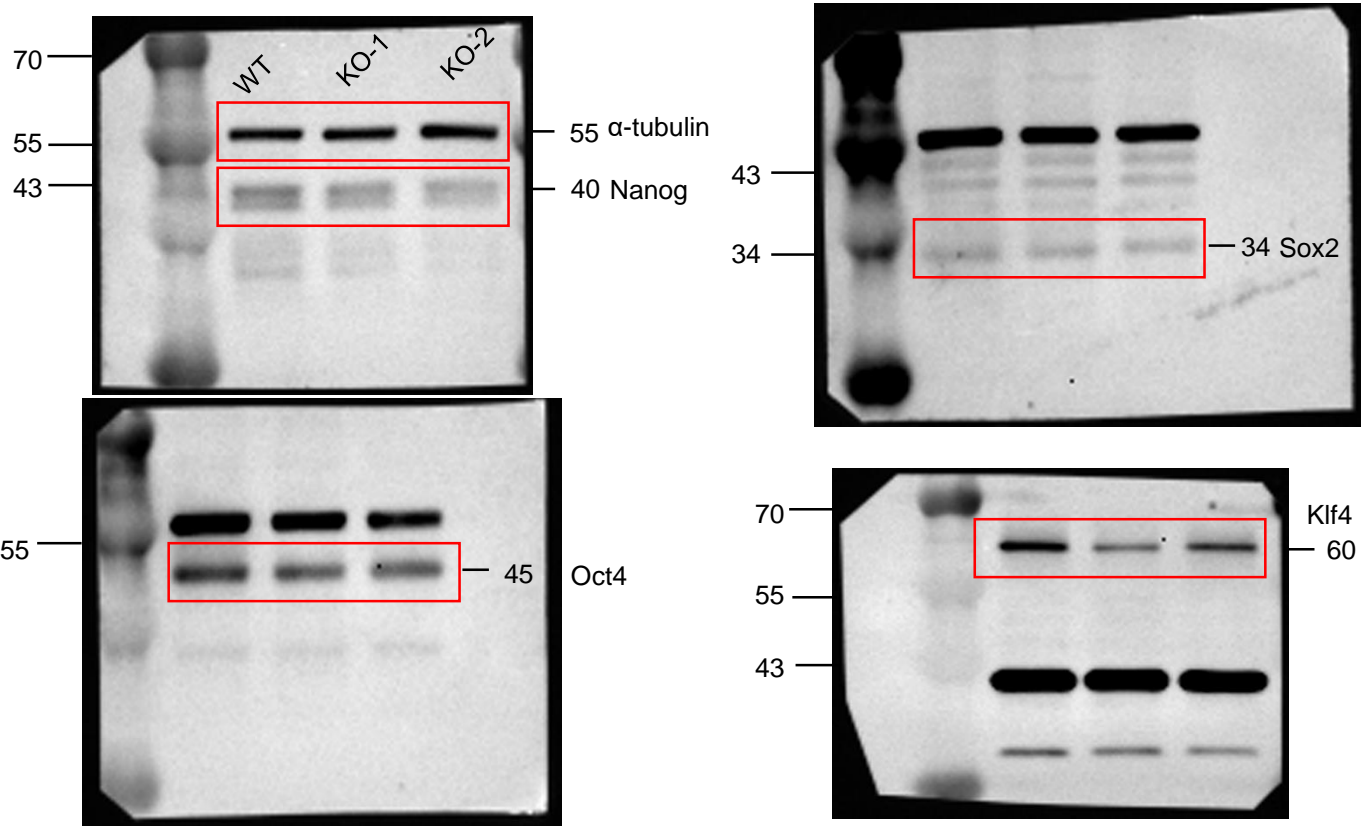

**Figure S3D**

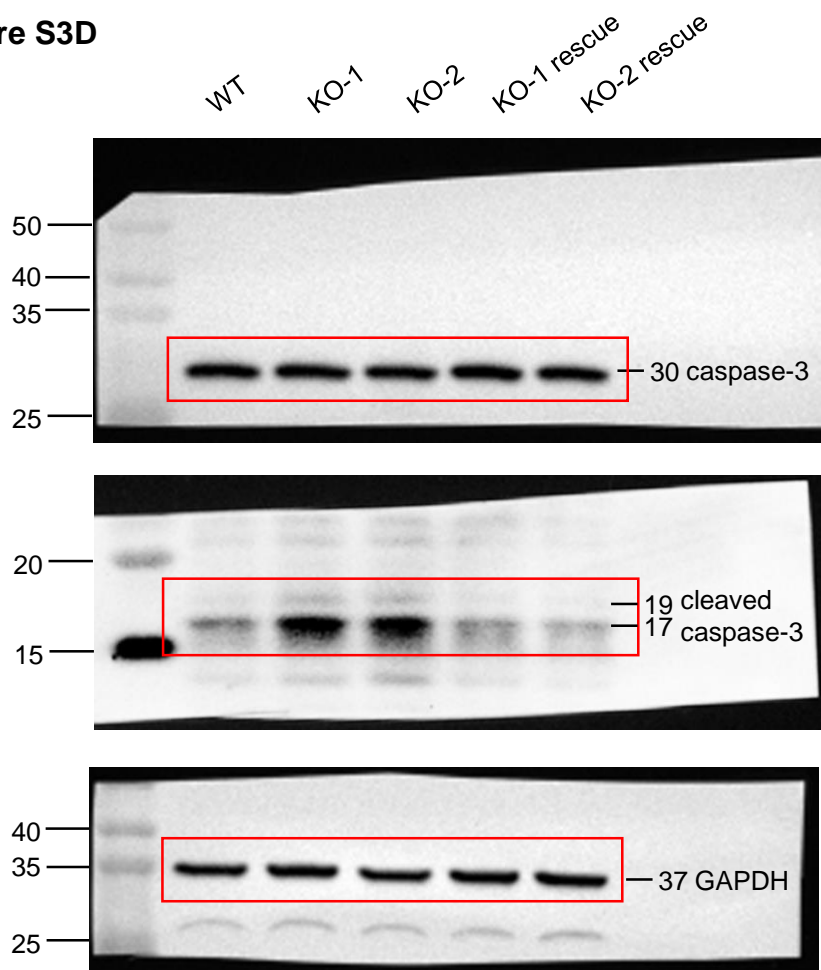

Figure S4E

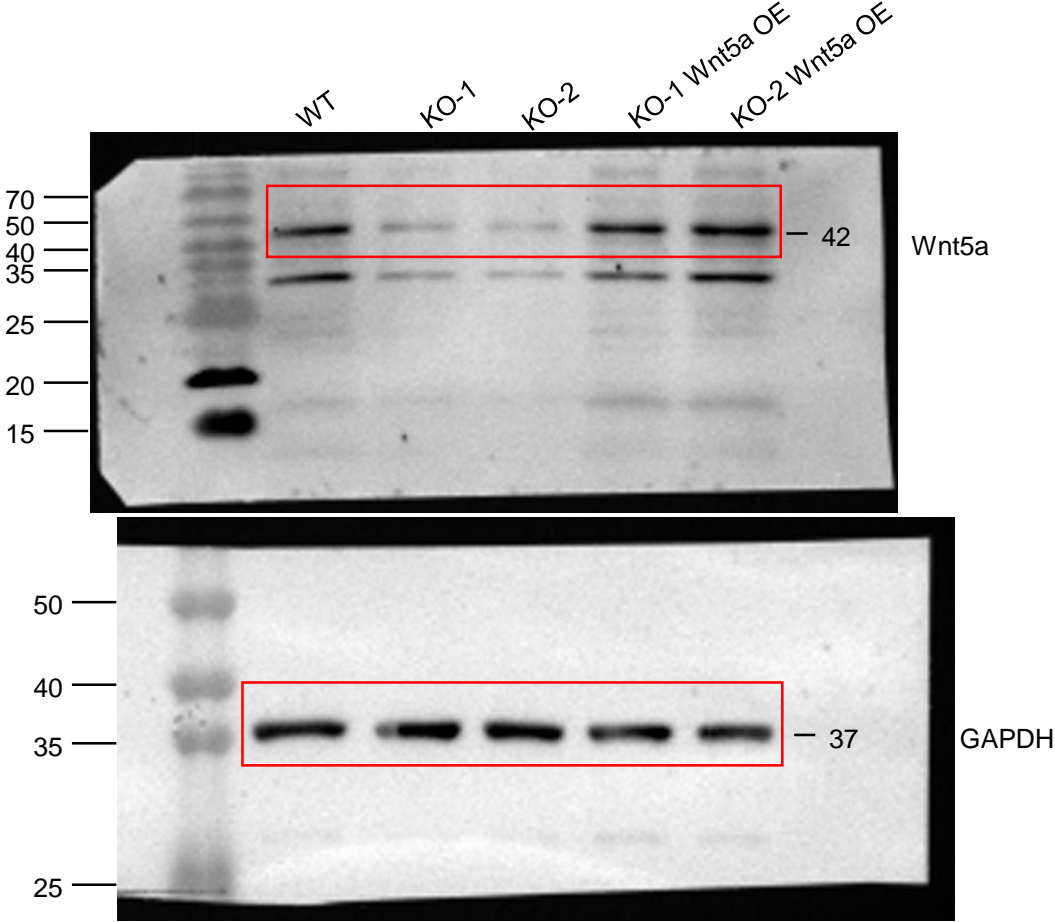

Supplement: Supplementary file 6 — Original Data File [file 41420_2023_1604_MOESM6_ESM.pdf]
