# Supplementary material for: Haspin balances the ratio of asymmetric cell division through Wnt5a and regulates cell fate decisions in mouse embryonic stem cells
Source: Cell Death Discov. 2023 Aug 23;9:307. doi: 10.1038/s41420-023-01604-w (PMC10447528; doi:10.1038/s41420-023-01604-w)
Supplement: Supplementary file 5 — Supplementary Figure Legends [file 41420_2023_1604_MOESM5_ESM.docx]

**Haspin Balances the Ratio of Asymmetric Cell Division through Wnt5a And Regulates Cell Fate Decisions in Mouse Embryonic Stem Cells**

**Supplemental Figure legends**

**Fig. S1 Effect of Haspin on cell cycle and proliferation in mESCs (related to Fig. 1).**

(A) Frequency of WT and Haspin-KO cells in the G1, S and G2/M phases. (*n* = 3). (B) The effect of WT and Haspin-KO on cell proliferation was determined by generating a proliferation curve (*n* = 3). (C) Protein levels of caspase-3 and cleaved caspase-3 in WT and Haspin-KO cells. (D) The statistical results of the protein expression in (C) (*n* = 3). The data in A, B and D are represented as the mean ± SEM. **p* < 0.05, ***p* < 0.01 and ****p* < 0.001.

**Fig. S2 Effect of Haspin knockdown on self-renewal and differentiation (related to Fig. 2).**

(A) The relative mRNA expression level of *Haspin* in groups transfected with Haspin negative control siRNA (H si-NC), siRNA-1 (H si-1) and siRNA-3 (H si-3) by qRT‒PCR. (B) Western blotting analysis of H3T3ph and H3S10ph in groups transfected with Haspin si-NC, si-1 and si-3. (C) The statistical results of the protein expression in (B) (*n* = 3). (D) Immunofluorescence of H3T3ph and H3S10ph in cells transfected with Haspin si-NC, si-1 and si-3. Scale bar: 50 μm. (E) Colony morphology and AP staining of cells transfected with Haspin si-NC, si-1 and si-3. Scale bar: 100 μm. (F) qRT‒PCR analysis of pluripotency genes expression in cells transfected with Haspin si-NC, si-1 and si-3 (*n* = 3). (G) qRT‒PCR analysis of three germ layer-specific markers in cells transfected with Haspin si-NC, si-1 and si-3 (*n* = 3). The data in A, C, F and G are represented as the mean ± SEM. **p* < 0.05, ***p* < 0.01 and ****p* < 0.001.

**Fig. S3** **Haspin is not essential for mESCs to maintain their pluripotency (related to Fig. 2).**

(A) Western blotting analysis and statistical results of the pluripotency protein (Nanog, Oct4, Sox2 and Klf4) expression in WT and Haspin-KO mESCs (*n* = 3). (B) Immunofluorescence and statistical results of pluripotency proteins in WT and Haspin-KO cells. *n* = 37 from three independent experiments. (C) Expression of three germ layer specific markers was examined by qRT‒PCR in WT and Haspin-KO mESCs that formed EB at Day 3 and Day 6 (*n* = 3). (D) Protein levels of caspase-3 and cleaved caspase-3 in WT, Haspin-KO and rescued mESCs that formed EB on Day 6. (E) The statistical results of the protein expression in (D) (*n* = 3). (F) Image of hematoxylin and eosin-stained sections of WT and Haspin-KO mESCs that formed teratomas, revealed that each teratoma included three germ layer tissues (black arrow). Scale bar: 200 μm. (G) qRT‒PCR analysis of differences in three germ layer specific markers between WT and Haspin-KO teratomas (*n* = 3). The data in A-C, E and G are represented as the mean ± SEM. **p* < 0.05, ***p* < 0.01 and ****p* < 0.001.

**Fig. S4 Transcriptome analysis of Haspin-KO mESCs (related to Fig. 4).**

(A) Heatmap illustration displaying gene expression of WT and Haspin-KO cells. (B) Gene Ontology (GO) enrichment analysis of DEGs in Haspin-KO cells compared with WT cells. (C) qRT‒PCR analysis was used to validate the RNA-seq data of genes related to the Wnt signaling pathway (*n* = 3). (D) The relative mRNA expression level of *Wnt5a* in WT, Haspin-KO and Wnt5a-RFP overexpression mESCs (*n* = 3). (E) The protein level of Wnt5a in WT, Haspin-KO and Wnt5a-RFP overexpressing mESCs. (F) The statistical analysis of Wnt5a protein expression (*n* = 3). (G) Phase-contrast microscopy images of WT, Haspin-KO and Wnt5a-RFP overexpressing mESCs. Scale bar: 100 μm. (H) Representative images of EB were obtained from WT, Haspin-KO and Wnt5a-RFP overexpressing mESCs on Day 3 and Day 6. Scale bar: 50 μm. (I) The area statistics of EB in (H). *n* = 20 for Day 3 and Day 6. The data in C, D, F and I are represented as the mean ± SEM. **p* < 0.05, ***p* < 0.01 and ****p* < 0.001.
